# Effects on the Profile of Circulating miRNAs after Single Bouts of Resistance Training with and without Blood Flow Restriction—A Three-Arm, Randomized Crossover Trial

**DOI:** 10.3390/ijms20133249

**Published:** 2019-07-02

**Authors:** Johanna Vogel, Daniel Niederer, Tobias Engeroff, Lutz Vogt, Christian Troidl, Thomas Schmitz-Rixen, Winfried Banzer, Kerstin Troidl

**Affiliations:** 1 Department of Sports Medicine, Institute of Sport Sciences, Goethe University, Ginnheimer Landstraße 39, 60487 Frankfurt, Germany; 2 Department of Experimental Cardiology, Medical Faculty, Justus-Liebig-University, 35392 Giessen, Germany; 3 Department of Cardiology, Kerckhoff Heart and Thorax Center, 61231 Bad Nauheim, Germany; 4 German Center for Cardiovascular Research (DZHK), Partner Site RheinMain, Frankfurt am Main, Germany; 5 Department of Vascular and Endovascular Surgery, University Hospital Frankfurt, Theodor-Stern-Kai 7, 60590 Frankfurt, Germany; 6 Institute for Occupational Medicine, Social Medicine and Environmental Medicine, University Hospital Frankfurt, Theodor-Stern-Kai 7, 60590 Frankfurt, Germany; 7 Department of Pharmacology, Max-Planck-Institute for Heart and Lung Research, Ludwigstrasse 43, 61231 Bad Nauheim, Germany

**Keywords:** circulating miRNA, miR-143-3p, blood flow restriction, peripheral artery disease, arteriogenesis, strength training

## Abstract

Background: The effects of blood flow restriction (training) may serve as a model of peripheral artery disease. In both conditions, circulating micro RNAs (miRNAs) are suggested to play a crucial role during exercise-induced arteriogenesis. We aimed to determine whether the profile of circulating miRNAs is altered after acute resistance training during blood flow restriction (BFR) as compared with unrestricted low- and high-volume training, and we hypothesized that miRNA that are relevant for arteriogenesis are affected after resistance training. Methods: Eighteen healthy volunteers (aged 25 ± 2 years) were enrolled in this three-arm, randomized-balanced crossover study. The arms were single bouts of leg flexion/extension resistance training at (1) 70% of the individual single-repetition maximum (1RM), (2) at 30% of the 1RM, and (3) at 30% of the 1RM with BFR (artificially applied by a cuff at 300 mm Hg). Before the first exercise intervention, the individual 1RM (N) and the blood flow velocity (m/s) used to validate the BFR application were determined. During each training intervention, load-associated outcomes (fatigue, heart rate, and exhaustion) were monitored. Acute effects (circulating miRNAs, lactate) were determined using pre-and post-intervention measurements. Results: All training interventions increased lactate concentration and heart rate (*p* < 0.001). The high-intensity intervention (HI) resulted in a higher lactate concentration than both lower-intensity training protocols with BFR (LI-BFR) and without (LI) (LI, *p* = 0.003; 30% LI-BFR, *p* = 0.008). The level of miR-143-3p was down-regulated by LI-BFR, and miR-139-5p, miR-143-3p, miR-195-5p, miR-197-3p, miR-30a-5p, and miR-10b-5p were up-regulated after HI. The lactate concentration and miR-143-3p expression showed a significant positive linear correlation (*p* = 0.009, *r* = 0.52). A partial correlation (intervention partialized) showed a systematic impact of the type of training (LI-BFR vs. HI) on the association (*r* = 0.35 remaining after partialization of training type). Conclusions: The strong effects of LI-BFR and HI on lactate- and arteriogenesis-associated miRNA-143-3p in young and healthy athletes are consistent with an important role of this particular miRNA in metabolic processes during (here) artificial blood flow restriction. BFR may be able to mimic the occlusion of a larger artery which leads to increased collateral flow, and it may therefore serve as an external stimulus of arteriogenesis.

## 1. Introduction

Arteriogenesis is defined as the growth of functional collateral arteries from pre-existing arterio-arteriolar anastomoses [1,2]. An initial trigger is the occlusion of a main artery, which occurs during peripheral artery disease (PAD). Such an occlusion redirects the blood flow to the pre-formed collateral arteries and thereby alters the fluid shear stress (FSS) [3]. The increased blood flow initiates vascular remodeling and diameter growth [4]. Several mechano-sensors and transducers that convey the FSS message during collateral remodeling have been proposed, including ion channels [5], the glycocalyx layer of endothelial cells (ECs) [6], nitric oxide (NO) [7], and microRNAs (miRNAs) [8].

These small, non-coding ribonucleic acids have been shown to play a decisive role in processes such as heart development, vascular regeneration, and tissue repair [9,10,11,12]. miRNAs are involved in post-transcriptional gene regulation by binding to mRNAs, causing the repression of translation and mRNA degradation, thus fine-tuning protein expression. Several miRNAs have been shown to control the response of vascular cells to hemodynamic stress [8]. In addition, miRNAs can be secreted and can thereby contribute to intercellular communication [13] or serve as circulating biomarkers [14]. 

Arteriogenesis can be amplified by exercise, as documented in human trials [15,16,17] and animal studies [18]. Therefore, according to international guidelines, PAD patients in Fontaine stage I or IIA/B (Rutherford 1–3) should be recommended for exercise training [19,20]. Mechanisms involved in the exercise-mediated benefits of treating PAD are thought to be the suppression of inflammation [1], expression of pro-inflammatory immune cells [21,22], and the improvement of endothelial function [23]. Beyond that, physical training has the potential to promote additional vascularization [24,25]. 

Comparable mechanisms have been discussed for the training effects of blood flow restriction exercises: Blood flow restriction training (BFR) is a resistance training method in which blood flow is reduced artificially. The decreased blood flow is usually caused by applying a blood pressure cuff at the origin of the extremity (arms or legs) to be trained. The mechanisms of BFR are thought to involve ischemic hypoxia and the increased expression of vascular endothelial growth factors [26]. The hemodynamic stimuli amplified by BFR (e.g., shear stress at the endothelium) lead to an increased release of the endothelial NO synthase, among other responses [27]. 

To achieve systematic effects during BFR, a lower resistance load is used than in classic resistance training without BFR: An intensity of 20% of the single repetition maximum (1RM) and a reduced training time of about 4–8 weeks have been demonstrated to have effects on muscle hypertrophy and muscular strength [28,29,30]. BFR training with a lower load in a shorter time can lead to the same results as resistance training with significantly higher loads (at 65% 1RM). In particular, increases in muscle thickness and strength are comparable between these strategies [31,32]. Due to the comparable effects and lower loads, BFR is of great relevance for training persons with physical limitations (e.g., patients with injuries, patients with cardiovascular diseases, or elderly persons) [33,34]. 

Despite the promising results derived from BFR as a method to mimic exercise effects under different occlusion conditions like PAD and the potential role of miRNAs as effectors after hemodynamic stress or FSS, nothing is known about the acute effects of strength training during BFR on miRNA levels. Therefore, this study was designed to determine whether the profile of circulating miRNAs is altered after resistance training during BFR, as compared with low- and high-volume training protocols with no BFR. Our hypotheses were: (1) Blood flow restriction leads to a reduced blood flow velocity; (2) low-intensity blood flow restriction training leads to metabolic responses that are similar to those of high-intensity strength training without blood flow restriction; (3) low-intensity blood flow restriction training and training without blood flow restriction lead to different expression characteristics of miRNAs.

## 2. Results

### 2.1. Sample

None of the participants withdrew their consent, and none had to be excluded. Eighteen healthy adults (females = 11; mean age 25 ± standard deviation (SD) 2 years; body mass index 22.1 ± 1.8 kg/m^2^) were included. 

### 2.2. Blood Flow Velocity

**Figure 1:**
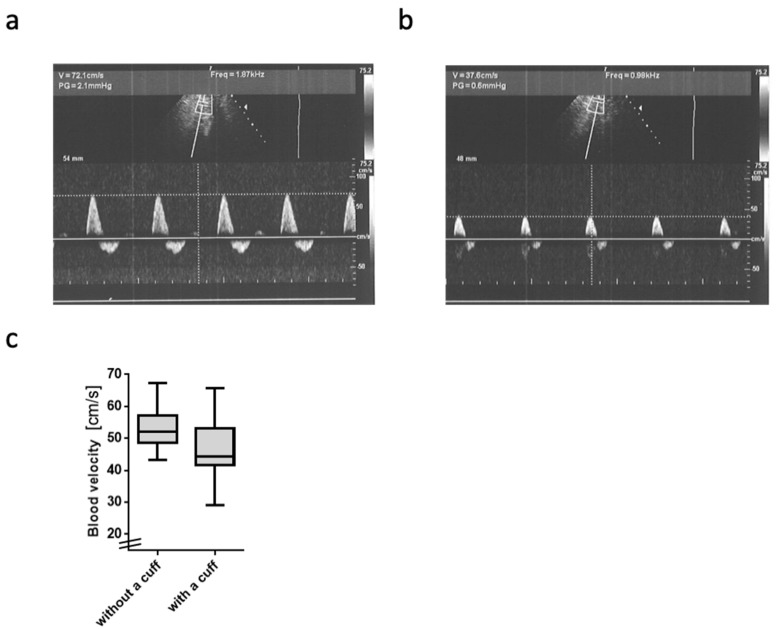
Blood flow velocity in the A. poplitea: (**a**,**b**) Representative original Doppler sonography blood flow profiles at rest: The upper images show the region of interest of the A.poplitea; the lower diagrams show the time course on the x-axis and the blood flow velocity heartbeat by heartbeat on the y-axis. (**a**) Blood flow velocity without wearing a cuff. (**b**) Blood flow velocity while wearing the cuff (occlusion pressure 300 mm Hg). (**c**) Boxplots of the grouped pre-post differences in blood flow velocity with and without cuff. Data are displayed as median and inter-quartile ranges plus range (whisker bars).

The blood flow velocity in the A. poplitea was significantly reduced by wearing the BFR cuff (compared to unrestricted, *p* = 0.002; mean intraindividual difference: −7.6 cm/s, −14%, Figure 1).

### 2.3. Basic Resistance Training Outcomes

#### 2.3.1. Objective Outcomes of the Training Interventions

**Figure 2:**
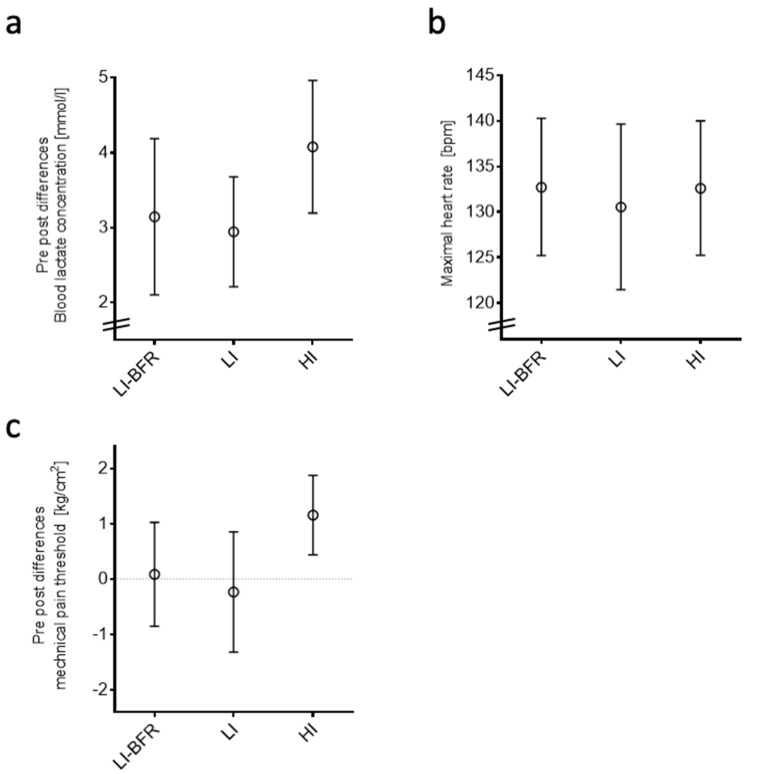
Objective outcomes of training interventions. Data are displayed as mean and 95% confidence intervals. Bpm, beats per minute; LI-BFR, low-intensity training with blood flow restriction; LI, low-intensity training; HI, high-intensity training. (**a**) Differences in blood lactate concentration pre- and post-training, (**b**) maximal heart rate, and (**c**) differences in mechanical pain threshold pre- and post-training.

Lactate concentration and heart rate were increased after all training interventions (*p* < 0.001) (Figure 2a,b). The lactate concentration was different between the groups: The high-intensity (HI) intervention resulted in a higher lactate concentration than both lower-intensity (LI) training protocols (LI, *p* = 0.003; LI-BFR, *p* = 0.008). In the HI group, the mechanical pain threshold increased from before to after training (*p* < 0.05) (Figure 2c).

#### 2.3.2. Participant-Reported Outcomes

**Figure 3:**
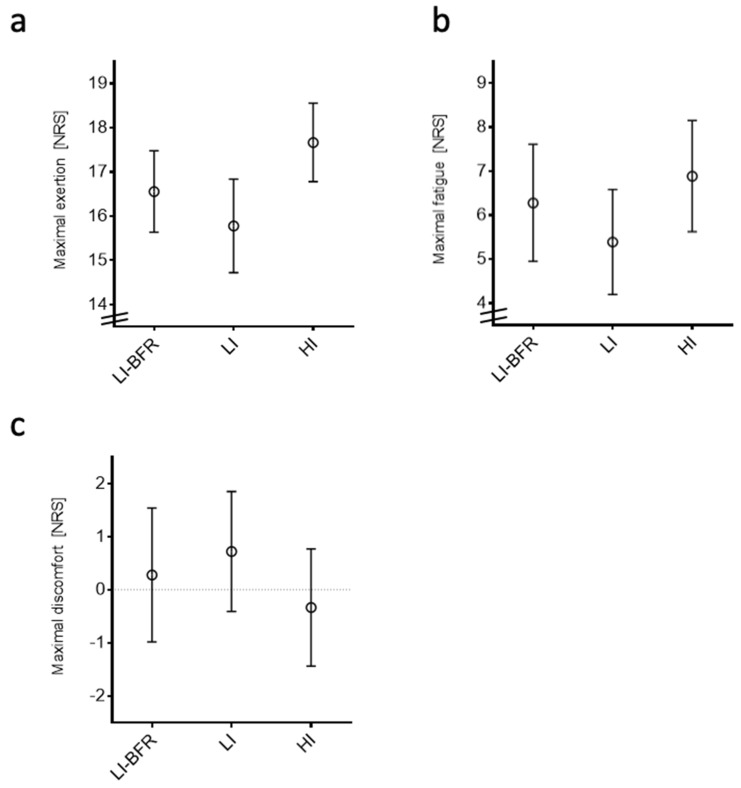
Participant-reported outcomes of training interventions. Data are displayed as mean and 95% confidence intervals. (**a**) Maximal exertion. (**b**) Maximal fatigue. (**c**) Maximal discomfort. NRS, numeric rating scale; LI-BFR, low-intensity training with blood flow restriction; LI, low-intensity training; HI, high-intensity training.

The perceived exertion was greater during the HI intervention than in the LI interventions (LI, *p* = 0.005; LI-BFR, *p* = 0.028). The HI group scored a lower value than the LI group in the feeling scale (*p* < 0.05). Participants in the LI group reported lower values on the fatigue scale than the LI-BFR group (*p* = 0.028) and the HI group (*p* = 0.004). The corresponding values are displayed in Figure 3.

### 2.4. Profiling of Circulating miRNAs

#### 2.4.1. Capillary Blood for miRNA Isolation, Expression Analysis, and Quantification

**Figure 4:**
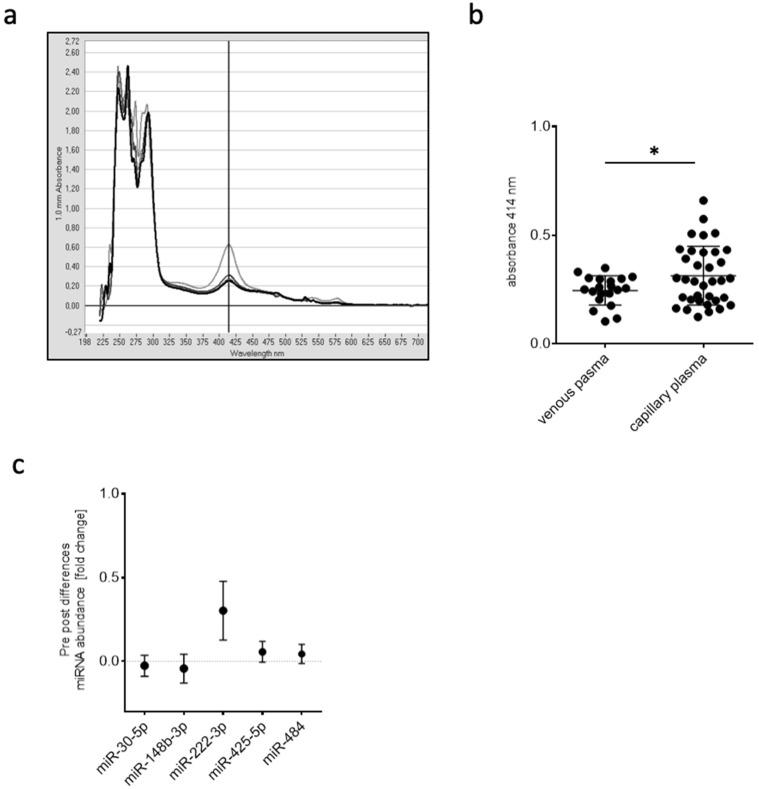
(**a**) Example of an OD scan from 200 to 700 nm with a distinct absorbance peak at 414 nm to assess hemolysis. Different lines depict different plasma samples. (**b**) OD_414_ values in plasma samples after venous or capillary blood draw (* *p* < 0.05). (**c**) Differences in miRNA abundance of typically detected miRNAs in plasma to determine stable expression pre- and post-training intervention.

A single capillary blood draw resulted in ≥50 µL plasma, and, in comparison to venous blood sampling, the average degree of hemolysis differed significantly (*p* < 0.05) as determined by OD_414_ in a pilot study (Figure 4a,b). Only 50 µL of plasma were sufficient to isolate total RNA and to reverse-transcribe miRNAs for real-time PCR-based quantification. In order to identify stably expressed reference genes for normalization, five candidate miRNAs (hsa-miR-30e-5p, hsa-miR-148b-3p, hsa-miR-222-3p, hsa-miR-425-5p, hsa-miR-484) were tested for stable expression over the entire range of samples being investigated. Except for hsa-miR-222-3p, all miRNAs were suitable for normalization (Figure 4c).

#### 2.4.2. Screening of Expression Changes in Circulating miRNAs before and after BFR Training 

**Figure 5:**
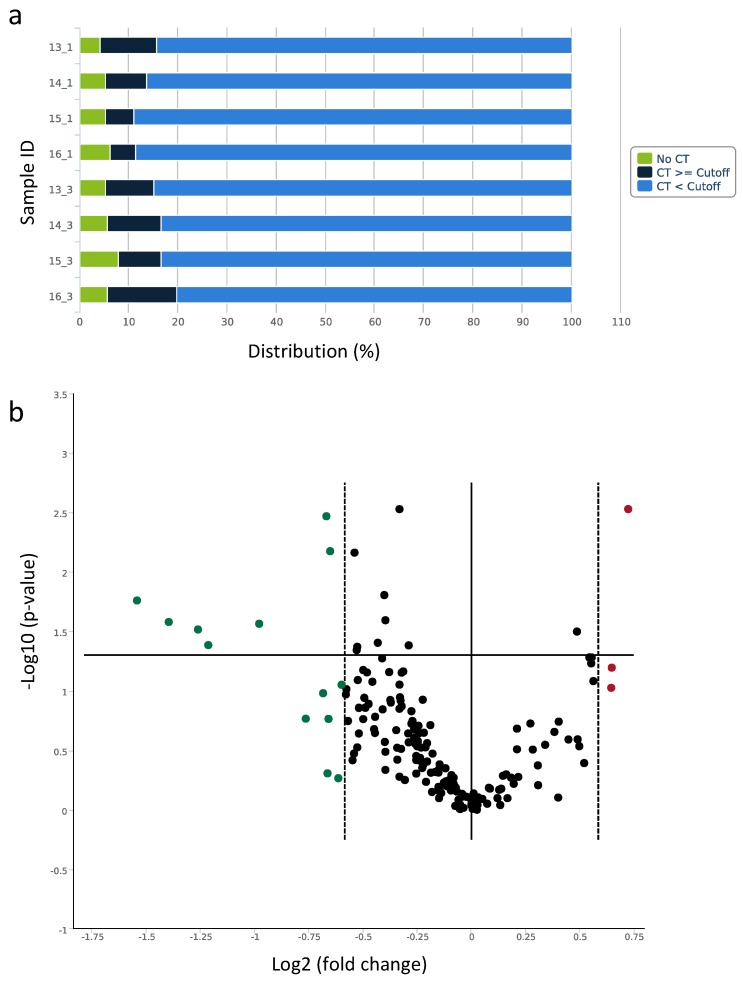
qPCR-based serum/plasma focus panel of circulating miRNAs (**a**) Distribution of *C*_t_ values for the processed data of each plasma sample. (**b**) Volcano plot of differentially expressed miRNAs pre- and post-LI-BFR training. Data points outside the two dashed lines are up-regulated (red) or down-regulated (green) more than x-fold. Data points above the solid horizontal line have *p*-values less than 0.05.

Based on the assumption that the LI-BFR reduces blood flow to the periphery in a way that is comparable to that of a PAD, eight plasma samples of four participants pre- (control) and post-training were selected and subjected to the human serum/plasma focus panel consisting of 179 miRNA assays targeting human plasma-relevant miRNAs, reference miRNAs, and spike-in controls. A global C_T_ mean of expressed miRNAs was used for normalization, and cel-miR-39-3p was included as an internal amplification control. In each sample, more than 80% of miRNAs surpassed the lower limit of detection of a *C*_t_ < 35 (Figure 5a). Significant miRNA expression changes were visualized in the volcano plot (Figure 5b). A total of 11 miRNAs were selected for further validation due to their markedly altered expression or previous association with collateral growth (Table 1). Interestingly, among the differentially expressed miRNAs identified, three arteriogenesis-associated, previously detected miRNAs were recovered: miR-143-3p, miR-195-5p, and miR-126-5p. 

#### 2.4.3. Analysis of miRNAs in Different Training Intervention Groups

**Figure 6:**
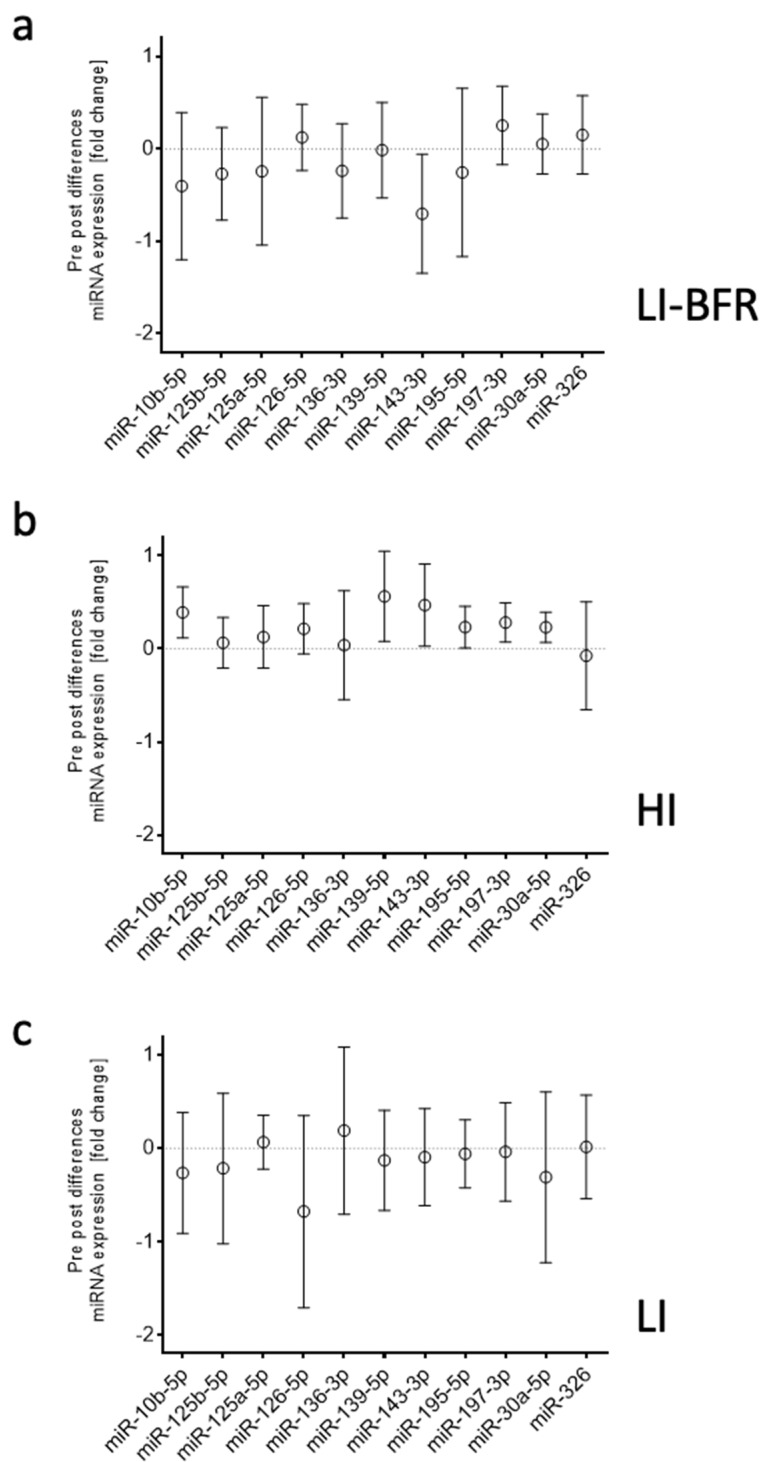
Differences in miRNA expression pre- and post-training. Data are displayed as mean and 95% confidence intervals. (**a**) LI-BFR, low-intensity training with blood flow restriction. (**b**) HI; high-intensity training. (**c**) LI, low-intensity training.

The abundance of these 11 differentially expressed miRNAs was analyzed in each training group in individual assays in a larger cohort of 12 participants. Only miR-143-3p was confirmed to be down-regulated after LI-BFR. In contrast to the initial screening results, miR-139-5p, miR-143-3p, miR-195-5p, miR-197-3p, miR-30a-5p, and miR-10b-5p were up-regulated after HI. There was no differential expression after LI. (Figure 6, Table 1)

### 2.5. Associations between Training Outcomes and Circulating miRNAs

**Figure 7:**
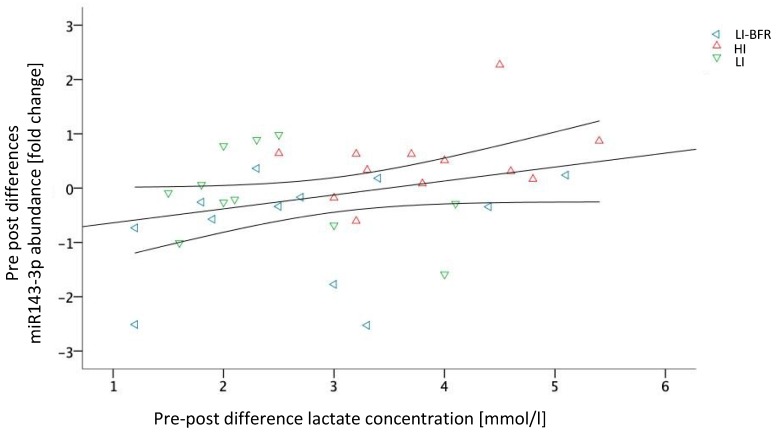
Scatterplot diagram of miR-143-3p and lactate concentration with a correlation line (including confidence intervals); LI-BFR, low-intensity training with blood flow restriction; HI, high-intensity training, LI, low-intensity training.

The pre-to-post changes in lactate concentration and miR-143-3p expression showed a significant linear positive correlation (intervention partialized) of *r* = 0.34 (*p* = 0.048). This correlation is visualized in Figure 7. Without considering the group as a partializing co-variate, lactate and miRNA-143-3p differences were associated with a coefficient of *r* = 0.305; however, this correlation lacks statistical significance (*p* = 0.075). No other systematic correlation between lactate concentration and miRNA expressions occurred.

## 3. Discussion

In this three-armed crossover study, we investigated the miRNA profiles in young, healthy athletes before and after various resistance training interventions (HI and LI). In addition, we employed peripheral blood flow restriction (LI-BFR) that was achieved by applying an external cuff during the resistance training at LI. We assumed that BFR can mimic the occlusion of a larger artery, leading to an increased collateral flow, and would therefore serve as an external stimulus of arteriogenesis.

The BFR application led to a decreased blood flow velocity in the popliteal artery, confirming our first hypothesis. The HI intervention showed the largest effects on lactate, and all interventions led to a comparable heart rate response. The LI intervention resulted in the smallest pre to post differences. Hypothesis 2 was thus partially verified (depending on the outcome). Our results further suggest that miRNA profiles were acutely affected by HI training and LI-BFR training but not by LI training alone. In particular, miR-143-3p expression correlated with training intensity, which verifies Hypothesis 3.

The fact that BFR application led to a decrease in the blood flow velocity (Hypothesis 1) confirms the validity of BFR. The reduction of 7.6 cm/s is far beyond the standard error of measurement found in inter-rater reliability analyses [35] and can thus be considered as clinically relevant. The mean blood flow velocity in the popliteal artery in PAD patients was recently found to be 41 ± 17 (SD) cm/s [36], which is considerably lower than the velocity we found in the no-cuff condition and is comparable to the one we found in the blood flow-restricted condition. Consequently, one may consider our model valid in terms of blood flow restriction and velocity, although the study population does not fully mimic the PAD caused by atherosclerosis.

All interventions induced increases in lactate and heart rate; the largest effects on lactate occurred after the HI intervention, whereas all interventions led to a comparable heart rate response. The LI intervention resulted in the smallest differences in pre- and post-intervention values in objective and participant-reported outcomes. The finding of a BFR-induced increase in lactate concentration is in accordance with previous reports [23]. The metabolic response thus varies with varying training intensity. If BFR training effects comparable to those of 70% 1RM sessions without BFR are to be reached, the current literature recommends BFR training at 30% of the individual 1RM [18]. Though we followed this recommendation, BFR elicited lower values/effects in some of the outcomes than during/after HI training; this is in contrast to our second hypothesis, which was only partially verified. One possible reason may be due to our sample. The majority of current studies adopting BFR training or interventions at 30% of the 1RM refer to an elderly or untrained population [18]. For a trained study population, a minority of studies in the literature suggest that 50% of the 1RM should be used to achieve a sufficient metabolic response [24]. 

We further analyzed the profiles of circulating miRNAs before and after resistance training. Our results of the quantification in each intervention group suggest that the profile of circulating miRNAs is altered as an acute effect of resistance training. In particular, we identified six miRNAs (miR-139-5p, miR-143-3p, miR-195-5p, miR-197-3p, miR-30a-5p, and miR-10b-5p) that are up-regulated after HI training. Only miR-143 was found to be down-regulated after LI-BFR training. The LI training, in contrast, had no systematic effect of either miRNA. Other studies also demonstrated both acute effects of intensive stimuli (miRNA-21, miRNA-146a, miRNA-221, and miRNA-222) and training effects (miRNA-146a, miRNA-222, and miRNA-20a) [37]. Consequently, we conclude that increased training intensity leads to increased miR-143-3p. The correlation of the lactate difference (BFR-LI, LI, and HI) and miR-143-3p abundance further indicates a decisive role depending on training intensity. In detail, 3.5 mmol/L of pre-to-post lactate difference was determined as a potential threshold from miR-143-3p down-regulation to its up-regulation. Consequently, a major share, but not only the lactate concentration (or the intensity of the training), is decisive for miRNA expression—as is (to a minor share) the type of training. More concretely, BFR seems not only to lead to lower lactate increases but also tendentiously leads to a down-regulation of miRNA-143-3p, whereas LI seems to be able to increase lactate concentration but does not affect miRNA-143-3p expression. The HI, in contrast, seemed to be able to both up-regulate lactate and miRNA-143-3p. Whether the differences between the conditions are due to the lower intensity or the type of training may be finally delineated by using the intensity increase up to 50% during BFR, as described above.

We have previously shown an association of miR-195-5p and miR-143-3p with collateral growth [38]. Both of these species were found to be highly up-regulated in the vascular tissue itself, and miR-143-3p was identified as an essential factor for proper collateral formation following femoral artery ligation in mice. The acute and local blockade of miR-143-3p in these mice completely abrogated arteriogenesis. In blood vessels, miR-143 is one of the most-studied miRNAs expressed by vascular smooth muscle cells, and, together with miR-145, this miRNA is thought to play a pivotal role in smooth muscle cell differentiation and vascular disease [8,13,39]. Furthermore, circulating miR-143-3p has been associated with cardiovascular disease [40] and is considered to be a predictor of aging and the acute adaptive response to resistance exercise [41]. 

Small volumes of capillary blood are routinely used for lactate diagnostics. We aimed to establish this routinely used, less invasive method of fingertip blood drawing for obtaining cell-free, non-hemolytic plasma samples suitable for the isolation and quantification of circulating miRNAs. Indeed, this method was successful in yielding plasma samples reproducible in quality and volume. All miRNAs identified, with the exception of miR-10b-5p, were validated in terms of independency of hemolytic score. For miRNA profiling, we included plasma samples of four participants with an OD_414_ < 0.3 and an increased lactate concentration after training intervention, a maximal heart rate during training of at least 60% of maximal calculated heart rate, and a participant-reported “very hard” intensity on the Borg scale of at least 16 points (data not shown).

Our results are in line with the proposed epigenetic potential of lifestyle interventions that may alter gene expression [42,43]. Therefore, we postulate that in order to maximize the beneficial role of miR-143-3p in collateral growth, training intensity will have to be adjusted. For symptomatic PAD patients, controlled training is an efficient, conservative therapy that is a good alternative to invasive therapies. The formation of collaterals and compensatory blood flow is the goal of conservative treatment. However, since the success of training varies, responders and non-responders must be identified. Thus, future studies are needed to (1) confirm our findings in PAD patients, (2) delineate the mechanisms of how miRNA-143-3p may be decisive in response or non-response to resistance training, and (3) determine how a pre-intervention screening of miRNA-143-3p or other microRNAs can be used to stratify responders and non-responders for the individualization of intervention/training goals.

## 4. Materials and Methods

### 4.1. Ethical Standard and Study Design

The study had a randomized-balanced crossover design. Ethical approval was obtained from the local institutional review board (protocol number 2018-16, 17.06.2018, Ethics Committee Department 5 Psychology and Sports Sciences Goethe-University Frankfurt). The trial was conducted in accordance with the ethical standards set down by the declaration of Helsinki (World medical Association) Declaration of Helsinki–Ethical Principles for Medical Research Involving Human Subjects) with its recent modification of 2013 (Fortaleza). All participants gave written informed consent prior to study enrollment.

### 4.2. Sample

Participants were considered eligible if they fulfilled the following criteria: (1) Healthy and (2) aged 18 to 30 years. Exclusion criteria comprised (1) severe psychiatric, neurological, or cardiovascular diseases; (2) acute orthopedic disorders; (3) pregnancy; (4) muscle soreness; and (5) intake of painkillers, analgesics, or muscle relaxants within the previous 48 hours. 

### 4.3. Experimental Design 

The experimental design incorporated three arms. Each participant performed each of the three conditions once (on three different days with a washout of at least 7 days in between) in a randomized, balanced sequence. Before the first exercise intervention, blood flow velocity to validate the BFR application and the individual 1RM from the knee extensor and knee flexor were determined. During each training intervention, loading-associated outcomes were monitored. Acute effects were determined using pre- and post-intervention measurements.

### 4.4. Blood Flow Velocity Measurement

The arterial blood flow velocity in the popliteal artery was measured in one leg (side randomly chosen, in the prone position) with and without external blood flow restriction caused/provoked by the BFR cuff (7 cm wide; nylon pneumatic cuff) at 300 mm Hg. Doppler sonography (Siemens Acuson X300, Munich, Germany) was used with spectral analysis, and data were gathered about components of the flow profile. The procedure is reliable [35].

### 4.5. RM Determination

Prior to the 1RM determination, participants warmed up by cycling for 5 min on a stationary bicycle. After a one-minute rest, an individualized starting load (~80% of estimated the 1RM, estimation based on sex, weight, and strength training experience) was moved through the full range of motion in sagittal plane (knee extension and flexion). After each successful performance, the weight increased until an attempt failed. One-minute rests were given between each attempt, and the 1RM was attained within a maximum of 5 attempts.

### 4.6. Intervention

The three training conditions were (1) resistance training during BFR at 30% of the 1RM, (2) resistance training without BFR at 30% of the 1RM, and (3) resistance training without BFR at 70% of the 1RM. The possible sequences of the conditions to be performed were randomly assigned to the participants in a balanced frequency. Resistance training comprised knee extensors and flexors on a combination training device (Schnell M3 Diagnosis, Peutenhausen, Germany). In conditions (1) and (2), resistance training with 30% of 1RM was adopted, and each exercise period consisted of 75 repetitions divided into 4 sets with a rest period of 90 s. In contrast, in condition (3), the resistance training was performed at 70% of the 1RM; here, 30 repetitions divided into 3 sets with a break of 90 seconds between sets. During condition (1), blood pressure cuffs inflated to 300 mm Hg were applied to both legs. The exercise protocols have been used previously in other studies and have been classified as low risk [44]. The washout phase between test days was at least 7 days. On each test day, a standardized control condition (“do-nothing” phase) was performed for 20 min before the test condition.

### 4.7. Assessments

#### 4.7.1. Laboratory Analytic Outcomes

##### Blood Lactate Concentration

Before and directly after each intervention, capillary blood was taken by pricking the earlobe with a safety lancet. The sample was applied directly to a test strip to determine lactate concentration (mmol/l) by means of a portable, hand-held unit (Lactate Scout, SensLab GmbH, Leipzig, Germany).

##### Heart Rate

During the intervention, a chest belt (Polar H7) and heart rate receiver (Polar M 430) continuously measured heart rate (beats/min). The maximum heart rate was selected for further analyses.

##### Mechanical Pain Threshold

Before and directly after each intervention, the mechanical pain threshold (N/cm^2^) was determined. Participants reclined supine on a bench with their legs extended. With an algometer (FPK, Wagner Instruments, Greenwich, CT, USA) pressure was applied on the skin (1 cm^2^). Three measurements were taken in the middle of both mm. recti femoris. The average of the three measurements was selected for further analyses.

##### Blood Sampling and Plasma Preparation for miRNA Profiling

In order to minimize pre-analytical variables that might influence the miRNA expression profile, care was taken in the collection of blood and the preparation of plasma to prevent blood cell contamination and hemolysis. Before and after each intervention, fingertip capillary blood samples (≥200 μL) were collected in microvettes (system for capillary blood collection) containing Ethylenediaminetetraacetic acid (EDTA). Blood samples were centrifuged for 10 min at 3000 rpm and 4 °C. After the first centrifugation step, the upper plasma phase was transferred to a new tube without disturbing the intermediate buffy coat layer. The plasma samples were centrifuged a second time for 10 min at 15,000 rpm and 4 °C. The cleared supernatant was carefully transferred to a new tube and frozen at −80 °C.

##### Determination of Hemolysis

To assess hemolysis, oxyhemoglobin absorbance was measured at 414 nm in plasma samples using NanoDrop (peqlab Biotechnologie GmbH; Erlangen, Germany).

##### miRNA Isolation

Sample amounts were standardized by volume: The same volume of plasma was used for each RNA isolation, and the same volume of purified RNA was used for all further analyses. The miRNAs were isolated from 50 µL (qRT-PCR) or 200 µL (PANEL screen) of plasma using a column-based protocol (miRNeasy Serum/Plasma Advanced Kit, (Qiagen, Hilden, Germany) according to the manufacturer’s protocol. cel-miR-39 from *Caenorhabditis elegans* (1 nM) was spiked in. In the final step, total RNA (>18 nucleotides) was eluted using 20 µl of RNase-free water.

##### Reverse Transcription and miRNA Profiling

For reverse transcription, the miRCURY LNA RT Kit (Qiagen, Hilden, Germany) was used. Undiluted complementary DNA (cDNA, 20 µL) was used for miRCURY LNA miRNA Focus Panel Human Serum/Plasma (YAHS-106Y) in the 2 × 96-well plate format. The Human Serum/Plasma Focus Panel includes 179 miRNA assays targeting relevant miRNAs, reference miRNAs, and spike-in controls.

##### Reverse Transcription and qRT-PCR

Following reverse transcription as described above, quantitative real-time PCR was performed using miRCURY LNA miRNA PCR assays (Appendix A) in a 10-µL reaction containing 3 µL of cDNA (1:30) and a CFX real-time PCR detection system (BioRad, Munich, Germany). Assays were performed in triplicate. The amount of the respective miRNA was normalized to miR-425-3p and cel-miR-39.

For the miRCURY miRNA PCR analysis, v1.0 raw *C*_t_ data from real-time PCR were up-loaded at (https://www.qiagen.com/us/shop/genes-and-pathways/data-analysis-center-overview-page). Cel-miR-39-3p was used as an internal spike-in amplification control. A *C*_t_ cut-off of 35 was set as the lower limit of detection. A global *C*_t_ mean of expressed miRNAs was used for normalization, and the fold change was calculated as (2^−ΔΔ*C*t^), which represents the average normalized miRNA expression (2^−ΔΔ*C*t^) of the samples in the test group divided by the average normalized miRNA expression (2^−ΔΔ*C*t^) of the samples in the control group.

#### 4.7.2. Self-Reported Outcomes

Self-reported outcomes consisted of rates of perceived exertion (RPE-Borg; Likert 6 to 20 point scale) [45], current well-being assessments (feeling scale: (+5 to −5, 10 point Likert scale)), and fatigue reporting (numeric rating scale NRS: 0 to 10 points). All self-reported parameters were assessed once after each intervention. The participants were asked to refer to the highest intensity during (RPE and feeling scale) or at the end (fatigue) of each intervention.

### 4.8. Data Analyses and Statistics

For all outcomes assessed before and after each intervention, real values and absolute pre-to-post differences were used for further analysis. Continuously assessed variables were processed in their real values.

After the following plausibility control, all analyses were performed based on the results of the initial checking for relevant underlying assumptions to test for parametric or nonparametric characteristics (data, distribution of the variances and variance homogeneity). Between-group differences and pre-to-post changes were assessed using omnibus and follow-up post-hoc testing. SPSS 23 (SPSS Inc., Chicago, IL, USA) and GraphPad software PRISM5 for Mac (GraphPad Software, La Jolla, CA, USA) were used to conduct all statistical calculations and create figures. An alpha-error level of 5% was considered to be a relevant cut-off value for significance testing, with *p*-values below 0.05 indicating significant differences.

Friedman tests were performed for omnibus between-group comparisons for all resistance training outcomes (or the a priori calculated differences). For significant omnibus testing, post-hoc comparisons using post-hoc Bonferroni–Holm tests and alpha-error-adjusted Mann–Whitney-U-tests were performed. For pre-to-post significance testing, Wilcoxon tests were performed.

To identify significant miRNA expression changes between conditions, a fold regulation was calculated, and a fold-change threshold of 1.5 was defined. Significant miRNA expression changes were visualized using the volcano plot. For each miRNA showing a significant expression change, a pairwise group comparison (Student’s *t*-test) was made based on the 2^−ΔΔ*C*t^ value of the replicate samples. The *p*-value calculation was based on a parametric, two-sample, equal variance, unpaired, and two-tailed distribution.

The potential associations between the kinematic (treatment) effects of the miRNA and lactate were analyzed using partial linear regression with the covariate group allocation.

## Figures and Tables

**Table 1:** Over-expressed miRNAs (fold regulation values greater than 1.5) and under-expressed miRNAs (fold regulation values less than −1.5) detected in the screen and analyzed in the three different training groups.

miRNA ID	Fold Change Screen	*p*-Value	HI	LI-BFR	LI
hsa-miR-197-3p	1.56	0.094			
hsa-miR-326	1.65	0.002 **			
hsa-miR-136-3p	1.57	0.063			
**hsa-miR-143-3p**	−1.7	0.170	up *	down *	
hsa-miR-30a-5p	−1.59	0.003 **	up **		
hsa-miR-139-5p	−1.97	0.027 *	up *		
hsa-miR-125a-5p	−2.63	0.026 *			
hsa-miR-375	−1.59	0.492			
hsa-miR-99a-5p	−1.58	0.171			
**hsa-miR-126-5p**	−1.57	0.006 **			
hsa-miR-10b-5p	−2.4	0.030 *	up **		
**hsa-miR-195-5p**	−2.92	0.017 *	up *		
hsa-miR-125b-5p	−2.32	0.041 *			
hsa-miR-100-5p	−1.61	0.104			
hsa-miR-362-3p	−1.52	0.088			
hsa-miR-376c-3p	−1.53	0.540			

* *p*-value < 0.05, ** *p*-value < 0.01, HI: High intensity training, LI-BFR: Low intensity training with blood flow restriction, LI: Low intensity training. Bold text shows the miRNAs that have been previously associated with collateral growth.

## Abbreviations

miRNA	microRNA
PAD	Peripheral artery disease
BFR	Blood flow restriction
FSS	Fluid shear stress
HI	High intensity
LI	Low intensity
LI-BFR	Low intensity with blood flow restriction

## Appendix A

**Table A1:** miRCURY LNA miRNA PCR assays for normalization.

hsa-miR-30e-5p	YP00204714	5′UGUAAACAUCCUUGACUGGAAG
hsa-miR-148b-3p	YP00204047	5′UCAGUGCAUCACAGAACUUUGU
hsa-miR-222-3p	YP00204551	5′AGCUACAUCUGGCUACUGGGU
hsa-miR-425-5p	YP00204337	5′AAUGACACGAUCACUCCCGUUGA
hsa-miR-484	YP00205636	5′UCAGGCUCAGUCCCCUCCCGAU

**Table A2:** miRCURY LNA miRNA PCR assays for screening hits.

hsa-miR-197-3p	YP00204380	5′UUCACCACCUUCUCCACCCAGC
hsa miR-326	YP00204512	5′CCUCUGGGCCCUUCCUCCAG
hsa-miR-136-3p	YP00205503	5′CAUCAUCGUCUCAAAUGAGUCU
hsa-miR-143-3p	YP00205992	5′UGAGAUGAAGCACUGUAGCUC
hsa-miR-30a-5p	YP00205695	5′UGUAAACAUCCUCGACUGGAAG
hsa-miR-139-5p	YP00205874	5′UCUACAGUGCACGUGUCUCCAGU
hsa-miR-125a-5p	YP00204339	5′UCCCUGAGACCCUUUAACCUGUGA
hsa-miR-125b-5p	YP00205713	5′UCCCUGAGACCCUAACUUGUGA
hsa-miR-126-5p	YP00206010	5′CAUUAUUACUUUUGGUACGCG
hsa-miR-10b-5p	YP00205637	5′UACCCUGUAGAACCGAAUUUGUG
hsa-miR-195-5p	YP00205869	5′UAGCAGCACAGAAAUAUUGGC
